# The effect of progressive muscle relaxation technique and myofascial release technique on premenstrual symptoms, blood circulation, and quality of life in women with premenstrual syndrome: A single-blind randomized controlled study

**DOI:** 10.1097/MD.0000000000034223

**Published:** 2023-07-07

**Authors:** Cisel Demiralp Ovgun, Emine Handan Tuzun

**Affiliations:** a Department of Physiotherapy and Rehabilitation, Eastern Mediterranean University, Famagusta, 99628 North Cyprus via Mersin 10, Turkey.

**Keywords:** myofascial release therapy, premenstrual syndrome, progressive relaxation

## Abstract

Premenstrual syndrome (PMS) is a common condition in women of reproductive age. It is characterized by a range of behavioral, physical, and psychological symptoms. The aim of this study is to investigate the effects of progressive relaxation and myofascial release technique (MRT) on premenstrual symptoms, blood flow rate, pain, sleep quality and quality of life, in women with PMS.

**Method:**

The study will conduct as a single-blind randomized controlled trial. Study registered at the ClinicalTrial.gov Protocol ID: NCT05836454. The volunteers will be randomized using allocation software to be divided into 3 groups: the progressive muscle relaxation group, the MRT group, and the control group. Assessments will be conducted by another physical therapist who is blinded to the groups. The assessments will include the Premenstrual Syndrome Severity Score, Blood Flow Measurements, Short Form McGill Pain Questionnaire, the Pittsburgh Sleep Quality Index and Short Form-36 Health Survey.

**Discussion:**

Since both methods provide relaxation, improve symptoms and quality of life, they have not been compared to each other in the literature. This prompt to us to plan this study.

## 1. Introduction

Premenstrual syndrome (PMS) is a common condition experienced by women of reproductive age. It is characterized by a range of behavioral, physical, and psychological symptoms that occur during the luteal phase of the menstrual cycle, typically 7 to 10 days before menstrual bleeding starts and ending with the onset of the secretory phase, due to hormonal changes. These symptoms can include cramps, changes in appetite, fatigue, irritability, edema, pain, sleep disturbances, depressive thoughts, and anxiety.^[1,2]^ The prevalence of PMS in Turkey has been reported as 52.2%, although it may vary by region. The prevalence also differs in other countries, such as Thailand (30%), Japan (9.9%), India (65%), Iran (39.9%), and Bulgaria (60%).^[3–6]^ With over 200 symptoms, PMS can be severe enough to disrupt normal daily activities and even lead to problems in personal relationships. It can cause loss of productivity, anxiety, depression, suicidal thoughts in women of reproductive age in severe cases, greatly reducing their quality of life.^[5–7]^ Additionally, PMS has been shown to have a negative impact on sleep quality, which is believed to be due to hormonal changes during the luteal phase. Compared to the follicular phase, there is less response to melatonin, dysregulation of the circadian rhythm, an increase in progesterone levels, and an increase in gamma-aminobutyric acid levels, which can contribute to mood and sleep disorders.^[8,9]^

Women use various methods to manage PMS symptoms, including physiotherapy, lifestyle changes, diet, stress management, cognitive behavioral therapy, alternative medicine, and pharmacological treatment.^[10]^ Exercise has been found to have a positive effect on both the physical and psychological symptoms of PMS. Research has shown that exercise can reduce muscle cramps, which are one of the physical symptoms of PMS, by increasing blood circulation.^[11,12]^

Progressive relaxation exercises (PRE) were first introduced by Jacobson in 1924. Relaxation has been shown to decrease oxygen consumption, muscle tone, heart rate, and respiratory rate, and can help patients shift their focus away from pain, potentially increasing endorphin production and reducing pain intensity.^[13–15]^ Today, PRE are used to treat a variety of conditions, including, cancer, chronic pain, post-surgical pain, chronic fatigue, stress and anxiety, and women health physiotherapy.^[16–19]^ In a randomized controlled study of women with PMS, PRE were found to lead to statistically significant reductions in depression and anxiety levels.^[20]^ Furthermore, the Benson relaxation technique has been shown to produce significant improvements in physical and mental symptoms, as well as oxidative stress markers, in women with PMS.^[21]^ Moreover, Ramaiah and Albokhary found that both progressive relaxation exercise and home exercise led to statistically significant improvements in menstrual pain intensity, possibly due to increased blood flow and inhibition of the sympathetic nervous system.^[22]^ Other methods used to cope with PMS include massage, aromatherapy, reflexology, and myofascial release technique (MRT).

MRT is a physiotherapy technique that aims to mobilize soft tissue, increase fascial mobility, reducing adhesions and pain.^[23,24]^ The technique involves applying slow and continuous pressure directly or indirectly to the fascia layers, which can result in the reordering of collagen and elastin fibers, allowing for optimal reorganization of the fascia layers and increased local circulation. MRT can be used to treat a variety of conditions, including subacute low back pain, fibromyalgia, lateral epicondylitis, plantar fasciitis, headaches, post-breast cancer fatigue, pelvic pain, and women health concerns.^[25]^ Studies have shown that MRT can increase acute lumbar paraspinal blood flow and venous blood flow in postmenopausal women with chronic venous insufficiency.^[26,27]^

A recent study compared MRT with connective tissue massage in women with primary dysmenorrhea and found both methods to be effective in reducing pain and increasing pain threshold. The increase in pain threshold and decrease in pain intensity may be attributed to MRT ability to relax the fascia, reduce sensitivity, and increase myometrial blood flow.^[28]^ However, to the best of our knowledge, there is currently no research available in the literature regarding the medium-long term effects of MRT in women with PMS. Furthermore, there has been no direct comparison between the effects of PRE and MRT in this population.

The aim of this study is to investigate the effects of PRE and MRT on premenstrual symptoms, blood flow rate, pain, sleep quality and quality of life in women with PMS.

## 2. Methods

The protocol is based on the Standard Protocol Items: Recommendations for Interventional Trials which provides a comprehensive framework for conducting interventional studies. (Appendix 1). This study registered in the ClinicalTrial.gov Protocol ID: NCT05836454. The study has been approved by the Easten Mediterranean University Health Sciences Ethics Sub-Board of Research and Publication Ethics Board (ETK00-2022-0045) and will adhere strictly to the principles set forth in the Declaration of Helsinki. Prior to participation, all subjects will receive a comprehensive verbal and written explanation from the researchers about the trial and will be required to provide voluntary written informed consent. Currently, this trial is in the participant recruitment stage.

### 2.1. Study design

**Figure 1.**
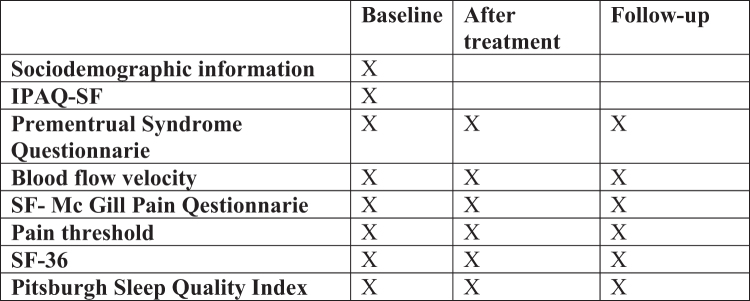
Assessment schedule.

The study is planned as a single-blind randomized controlled trial. Participants who meet the inclusion criteria were randomly assigned into 3 groups by the 1 block simple randomization method using Random Allocation Software by an expert statistician who is blinded. 31 women were assigned to the MRT group, 32 women to the PRE group, and 31 women to the control group. The evaluations will be performed by another physiotherapist who is blind to the group allocation. The evaluator has sufficient knowledge about all data collecting tools. All evaluations will be performed 1 week before the estimated start date of the menstrual cycle. The intervention will be applied for 2 menstrual cycle, 2 times a week, and each session will last about 20 to 30 minutes. Participants will inform that if they desire, another treatment method could be apply at the end of the study. Additionally, the session hours will be arranged to be compatible with their university class schedules, as they are university students. The assessments will be made at baseline, post-treatment and after follow-up period (Fig. 1). The data will be analyzed by using SPSS software (version 25.0). To improve data quality, data entry will be double-checked. The data file will be stored both in hardware and software formats. To ensure data security, the data will not be shared with third parties.

### 2.2. Subjects

The study population includes women aged 18 to 30 who are studying at the Faculty of Health Sciences of Eastern Mediterranean University. The sample size is calculated as follows: with F = 0.40, Alpha = 0.05, Beta = 0.90 and 3 groups, the initial sample size was calculated as 82 according to the assumptions of One-Way ANOVA test. Considering the dropout rate, this sample size is increased by 15% and the final sample size is calculated as 94.

#### 2.2.1. Inclusion criteria.

A score of 111 or higher on the PMS Scale,^[29]^

Experience of premenstrual symptoms for at least 3 months,

Having a pain score of at least 4 on the Visual Analog Scale during any menstrual period within the last 3 months.

Regular menstrual cycle for 12 months (24–35 days),

A weekly MET value of 600 MET-min/week or lower according to the International Physical, Activity Questionnaire- Short Form (IPAQ-SF).

#### 2.2.2. Exclusion criteria.

Individuals who underwent surgery in the last 6 months,

Individuals with musculoskeletal problems,

Individuals with chronic diseases,

Individuals receiving hormone therapy,

Pregnant individuals,

Individuals with urinary, genital, or gastrointestinal disorders,

Individuals who underwent hysterectomy surgery.

### 2.3. Interventions

#### 2.3.1. Progressive relaxation exercises.

The session will begin with 3 deep breaths and then proceed to the relaxation exercise. The muscles of the hand, arm, face, shoulder and neck, chest, abdomen, back, thigh and hip, legs and feet will be contracted and relaxed in sequence. The contraction time is 5 seconds (s), and the relaxation time is 10s. After all the muscles have been contracted and relaxed in sequence, whole body will be contracted and relaxed simultaneously with breathing. During the session, participants will ask to close their eyes try avoiding negative thoughts and get relax.^[30,31]^

#### 2.3.2. Myofascial release technique.

The technique will be done with dry hands without using any lubricating agent. In the treatment program, 3 minutes of tension will be applied to the superficial fascia, transversalis fascia and extraperitoneal fascia for antero-lateral release. Then, 3 minutes of tension will be applied to the thoracolumbar fascia and erector spinae muscle for posterior abdominal wall release. Additionally, 3 minutes of tension will be applied to the cervical region and posterior.

#### 2.3.3. Control group.

Individuals in the control group will be asked to continue their normal daily lives but no treatment will be administered.

### 2.4. Data collection tools

#### 2.4.1. Socio-demographic information form.

Age, body mass index, backgrounds, and educational status will be recorded. Also, characteristics of menstrual cycle like length of cycle and duration of bleeding will be recorded.

#### 2.4.2. Physical activity level.

The International Physical Activity Questionnaire- Short Form (IPAQ-SF) will be used to determine whether the inclusion criteria of meeting the physical activity level or not. The Turkish validity and reliability study conducted by Saglam et al will be used to evaluate the individuals’ duration of low, moderate, and high-intensity activity over the past week, resulting in an average energy expenditure value in kcal/week.^[32]^

#### 2.4.3. Premenstrual syndrome severity.

To evaluate the severity of premenstrual symptoms in participants, the Premenstrual Syndrome Assessment Scale developed by Gençdogan will be used. Premenstrual Syndrome Assessment Scale is a 44-item 5-point Likert-type scale. In the scoring of the scale, the “None” option is 1 point, the “Very little” option is 2 points, the “Sometimes” option is 3 points, the “Often” option is 4 points, and the “Constant” option is 5 points. The scale includes 9 subheadings: depressive feelings, anxiety, fatigue, irritability, depressive thoughts, pain, appetite changes, sleep changes, and swelling. Obtaining the half of maximum sub heading score is used to determine whether symptom exist in the individual. Participants with a total score of 111 or higher are considered to have PMS.^[33]^

#### 2.4.4. Evaluation of blood flow velocity.

Blood flow velocity will be evaluated using the Bestman Vascular Doppler BV520T. Measurements will be taken through the superior and inferior epigastric arteries, which provide blood flow to the abdominal wall and abdominal muscles. The superior epigastric artery can be found just medial to the midclavicular line, which is an imaginary line that runs vertically down the center of the chest, passing through the midpoint of the clavicle. The artery is located approximately 2 to 3 cm lateral to the xiphoid process, which is the small, cartilaginous protrusion at the bottom of the sternum. To locate the superficial inferior epigastric artery, the ultrasound probe is placed just above the midpoint of a line connecting the anterior superior iliac spine and the pubic tubercle. The anterior superior iliac spine is a bony prominence on the front of the hip bone, while the pubic tubercle is a small bony projection located on the pubic bone. The probe is angled inwards towards the midline of the body, and the superficial inferior epigastric artery can be visualized as a small pulsating vessel on the ultrasound screen. After applying ultrasound gel to the measurement area, the probe will be placed at a 30° angle and the measurement will be taken.^[26,27]^

#### 2.4.5. Evaluation of pain characteristics.

The Short Form McGill Pain Questionnaire will be used to evaluate pain. The questionnaire, which evaluates different aspects of pain: sensory pain (11 items) and affective pain (4 items), and consists of a total of 15 items. The 4-point likert-type scale is scored between 0 and 3 (0: none, 3: severe). The total pain score ranges from 0 to 45. The Turkish validity and reliability study of the questionnaire was conducted by Yakut et al in 2007.^[33]^

#### 2.4.6. Pain threshold.

Pain threshold will be evaluated using the Force Dial FDK 20 algometer is a handheld device manufactured by Wagner Instruments. Pressure will be applied to the body of the muscle to be evaluated until pain is felt. Participants will be asked to report when they first feel the pressure as pain, and the value on the algometer will be recorded in kg. Evaluation areas: Vastus medialis, Pectoralis major, Gluteus maximus, Gluteus medius, Iliopsoas, Lumbar paraspinalis, Trapezius muscles.^[34,35]^

#### 2.4.7. Sleep quality.

Sleep quality can be assessed through a variety of methods, including self-report questionnaire like the Pittsburgh Sleep Quality Index. Pittsburgh Sleep Quality Index consists of 19 items, evaluates the individual sleep quality, sleep latency, sleep duration, habitual sleep efficiency, sleep disturbances, use of sleeping medication and daytime dysfunction. The scale scores between 0 and 3 (0: no difficulty, 3: severe difficulty). The Turkish validity and reliability study of the scale was conducted by Ozturk et al in 2009.^[36]^

#### 2.4.8. Health-related quality of life.

HRQOL will be evaluated using the Short Form-36 Health Survey. The Turkish validity and reliability study was conducted by Kocyigit et al. The scale, which consists of 36 items and 8 sub-dimensions, has sub-dimensions of: physical function, social function, role limitations due to physical function, role limitations due to emotional problems, mental health, vitality, pain and perception of general health. In the evaluation of the scale, each sub-dimension scores between 0 and 100 points, 0 points indicating poor quality of life in the dimension and 100 points indicating good quality of life.^[37,38]^

### 2.5. Discussion

Women of reproductive age utilize various coping methods to effectively manage PMS. PRE are commonly used in women health and are regarded as an important method for managing PMS symptoms. These exercises have been found to induce positive changes in depression and anxiety. Additionally, the increased blood flow and elimination of substances such as prostaglandins from the area can play a crucial role in reducing spasms in the uterus and alleviating pain in the abdomen and pelvic region. It is also believed that PRE may exert inhibitory effects on the sympathetic nervous system.^[21,22]^

MRT has gained frequent usage in recent times and has been explored in the context of women with dysmenorrhea and PMS in the literature. This technique is known to raise the pain threshold and decrease pain intensity. It is believed that the improvements in pain management occur due to the relaxation of the fascia, which reduces sensitivity, and the subsequent increase in blood flow.^[23,24]^

Both methods offer relaxation, increased blood flow, and improved sleep quality and quality of life. Furthermore, these approaches have not been compared to each other in the literature, which prompted us to plan this study.

## Author contributions

**Conceptualization:** Cisel Demiralp Ovgun, Emine Handan Tuzun.

**Methodology:** Cisel Demiralp Ovgun, Emine Handan Tuzun.

**Project administration:** Cisel Demiralp Ovgun, Emine Handan Tuzun.

**Resources:** Cisel Demiralp Ovgun.

**Supervision:** Emine Handan Tuzun.

**Writing – original draft:** Cisel Demiralp Ovgun.

**Writing – review & editing:** Cisel Demiralp Ovgun, Emine Handan Tuzun.
